# Fears and uncertainties of expectant mothers during the COVID-19 pandemic: trying to reclaim control

**DOI:** 10.1080/17482631.2021.2018773

**Published:** 2022-01-10

**Authors:** Eman A. Abu Sabbah, Sondos B. Eqylan, Dua’ Yousef Al-Maharma, Fida Thekrallah, Reema R. Safadi

**Affiliations:** a Department of Maternal and Child Health Nursing, The University of Jordan, Amman, Jordan; b Emergency Department, Al-Hussein Hospital, Salt, Jordan; c Department of Obstetrics and Gynecology, Faculty of Medicine, The University of Jordan, Amman, Jordan

**Keywords:** COVID-19, expectant mothers, Jordan, lockdown, perinatal care, prenatal experience, qualitative research

## Abstract

**Purpose:**

The novel coronavirus disease (COVID-19) outbreak has exponentially spread across the globe and formed one of the greatest health threats in history. Pregnant women are vulnerable, and their vulnerability is amplified by the associated containment measures of the pandemic. In this study, we aim to explore and describe expectant mothers’ experiences during the lockdown of COVID-19.

**Method:**

A qualitative descriptive design was used. Eighteen pregnant and postpartum women were recruited through purposive and snowball sampling. Semi-structured phone call interviews were conducted by a female researcher. Braun and Clarke’s thematic data analysis was followed.

**Results:**

Three main themes are developed: (1) Living with fears and uncertainties amid the COVID-19 pandemic, (2) Lockdown disrupting the normalcy of pregnancy, (3) Trying to control the chaos of life. Five subthemes underlined the three themes.

**Conclusion:**

Findings portrayed expectant mothers’ uncertainties, fears, and the fragility of the healthcare systems in responding to mothers’ needs during the COVID-19 pandemic. Although the pandemic has resulted in disruption of the normalcy of pregnancy, mothers sought adaptive means to protect themselves and maintain control. Governmental authorities and healthcare providers are directly responsible to maintain considerate perinatal care services for expectant mothers during lockdown and crises.

## 1. Introduction

The novel coronavirus disease (COVID-19) outbreak that started in Wuhan, China at the end of 2019 has exponentially spread across the globe and formed one of the greatest health threats in history. The World Health Organization (WHO) declared COVID-19 as a pandemic where millions of people were infected (World Health Organization [WHO], 2020). The COVID-19 pandemic has affected all population strata but older adults, children, people with comorbidities or disabilities, and pregnant women and new mothers were severely affected (Ellington et al., 2020; Kotlar et al., 2021). The devastating impact of COVID-19 among older adults were not limited to a higher risk of mortality and severe disease process, but also added to their social isolation and mental health problems (United Nations, 2020a). Discrimination in care provision, disruption of social networks, and the enforced limited access to essential care services to control COVID-19 transmission further deteriorates older adults’ health conditions (Lekamwasam & Lekamwasam, 2020; United Nations, 2020a). As for children, the current evidence indicates that they are less likely to develop severe symptoms or die from COVID-19 (Centers for Disease Control and Prevention, 2021), however, the harmful effects have threatened children’s mental well-being, learning opportunities, social development, and safety (United Nations, 2020b). In addition, recent evidence shows that pregnant women have a higher risk for more severe illness from COVID-19 compared with non-pregnant women (American College of Obstetricians and Gynecologists, 2021; Ellington et al., 2020).

The scientific evidence has established an association of pregnancy and the risk for mental health problems due to hormonal, psychosocial, and familial role changes (Araji et al., 2020; Dunkel Schetter & Tanner, 2012). Systematic reviews show that antepartum anxiety and depression are prevalent among pregnant women with a global prevalence of 22.9% and 15–65%, respectively (Dadi et al., 2020; Dennis et al., 2017). Pregnant women are usually subject to worries and fears about their pregnancy and its outcome. Concerns regarding the unpredictability of childbirth, anticipated medical interventions, or complication during birth (Araji et al., 2020) led women to experience additional fears and exacerbated anxiety levels at the end of pregnancy. Additionally, the current evidence indicates that maternal wellbeing and foetal development are negatively influenced by maternal mental health problems. More specifically, anxiety, depression, and stress during pregnancy are associated with maternal and neonatal complications such as prematurity, low birth weight, disrupted parenting and bonding, and developmental problems during the child’s life (Araji et al., 2020; Dunkel Schetter & Tanner, 2012).

Beside the inevitable bio-psychological changes that occur during pregnancy, the environmental stressors (i.e., disaster/ pandemic) have an additional adverse effect on maternal mental wellbeing. Pregnant women’s vulnerability is amplified by the COVID-19 pandemic and the associated containment measures. In Jordan and everywhere else, the “Stay at Home” orders placed pregnancy and childbirth in radically new and unusual circumstances that jeopardized the physical and psychological wellbeing of mothers. During the chaos of this pandemic, anxiety, depression, fear, and post-traumatic symptoms were reported at a higher rate among expectant mothers (Ogunbiyi, 2021).

The literature about the pandemic has reported multiple factors that increased health concerns among expectant mothers. These factors include infection transmission vulnerability, inadequate prenatal care (PNC), inability to access reliable information, and social isolation from support networks (Mizrak Sahin & Kabakci, 2021; Mortazavi & Ghardashi, 2021; Ogunbiyi, 2021). Furthermore, the conflicting and rapidly changing messages on social media has also created high levels of fear, uncertainty, and anxiety among pregnant women (Mizrak Sahin & Kabakci, 2021; Mortazavi & Ghardashi, 2021). As a consequence to the emerging COVID-19 pandemic, enormous modifications in the provision of maternal healthcare services, such as turning to telemedicine, virtual perinatal healthcare, shorter hospitalizations, limiting birth companions, and postponing non-essential surgeries or procedures were ensued (Kotlar et al., 2021).

In Jordan, as in other parts of the world, stringent precautionary measures were adopted by government authorities to control the spread of COVID-19 and its adverse outcomes. By activating the National Defence Law—that is usually applied in time of war and crisis, a complete countrywide lockdown was enforced at March 21–25th, 2020 after declaring the state of emergency; it was described as one of the strictest in the world (Picheta & Qiblawi, 2020). Police and armed forces were allocated to main cities’ entrances to enforce the implementation of the lockdown and track violations. During this period of strict 24-hour curfew, only healthcare providers (HCPs) and vital sectors’ personnel were allowed to move on the streets; medical emergencies were handled through Civil Defence directorates (National Center for Security & Crises Management, 2020). In Jordan, the Civil Defence directorate undertakes the responsibility of providing services to protect citizen’s lives and properties and ensures their safety in health emergencies and natural disasters. One of the main and vital duties of the Civil Defence is the provision of rapid ambulance and healthcare service that is similar to the “Paramedic Services” in the United States. As of 26 March 2020, the curfew hours’ duration was reduced to become from 6:00 p.m. until 10 a.m. the following day. During the day time, people were allowed to walk (no cars) in their own neighbourhood to shop at local stores while strictly keeping physical-distancing guidelines and wearing face masks and gloves. At the end of April 2020, people could drive their cars on alternating days for an even and odd number of car plates and restricted to two passengers only. During this period of driving car restrictions, people had to obtain an electronic permit for partial movement within the same Governorate by applying to a specific platform to use their own car in case of emergency, such as in a health condition urgency, joining funeral rituals, or domestic crises.

In Jordan, health care services are provided at primary, secondary, and tertiary level through private and public health institutions. Public healthcare centres and hospitals are operated by the Ministry of Health, the Royal Medical Services, and the University Hospitals. Ever since the establishment of primary and maternal and child healthcare services in the late 1970s—1980s, healthcare sectors have stressed the importance of PNC that is widely offered in the public and private sectors (World Health Organization & The United Nations Children’s Fund, 1978). According to Jordan’s Population and Family and Health Survey (JPFHS) 2017–18, 98% of women aged 15–49 of all socioeconomic backgrounds received PNC from a skilled provider (i.e., obstetricians, nurses, midwives) for their most recent birth within the 5 years preceding the survey (Department of Statistics (DOS) and ICF, 2019). Approximately, 79% of pregnant women have received at least seven PNC visits out of 12 to13 recommended visits. Standard PNC services include iron supplements and protection against neonatal tetanus besides the regular check-ups for blood pressure and blood glucose measurements. Having had PNC highly emphasized and promoted by the health authorities in the community during the last three decades, this has become part of women’s pregnancy rituals and only under crucial circumstances they would miss these appointments. During the COVID-19 pandemic, this conventional service in outpatient clinics, including maternal and child healthcare centres, was abruptly halted. It took around ten weeks of closure before the curfew hours were slightly released, and services resumed. This led to a significant drop in the percentage of women receiving any form of maternity care including that required for serious pregnancy complications (Muhaidat et al., 2020).

In Jordan, most of the published reports were focused on assessing the public knowledge and attitude towards COVID-19 and the impact of lockdown as a preventive measure (Elayeh et al., 2020; Sallam et al., 2020). There is only one quantitative study by Muhaidat et al. (2020) that tackled Jordanian pregnant women’s concerns during the lockdown. Worldwide, based on our most recent literature search, only a limited number of studies have delved into exploring pregnant women’s feelings, concerns, and the emotional status during pregnancy from a qualitative standpoint (Mizrak Sahin & Kabakci, 2021; Mortazavi & Ghardashi, 2021). Therefore, this study aims to explore pregnant women’s experiences during the lockdown of COVID-19.

The current qualitative study will enable HCPs to understand expectant mothers’ feelings and concerns from an emic perspective. Therefore, the findings are expected to guide policy makers and HCPs in developing strategic plans for emergency situations that are considerate of expectant mothers’ needs during a pandemic. Furthermore, this study provides a deeper and holistic understanding of pregnant women’s health issues and needs that is complementary to quantitative studies that are focussed on statistical figures and bio-physiological factors and consequences. Qualitative studies that target pregnant women’s experiences during the pandemic are limited compared to those of quantitative nature either internationally or nationally. Therefore, we devote this study to understand the experience of expectant mothers’ concerns and needs during COVID-19 pandemic.

## 2. Methods

### 2.1 Design

A qualitative descriptive research design was used to describe expectant mothers’ experience during the lockdown of COVID-19. This approach is appropriate to explore the specific experience during a limited time or context for an in-depth understanding of pregnant mothers lived experience of a phenomenon to a possible description of the whole experience (Creswell, 2013). The terms expectant mothers and participants are used interchangeably. This study was guided by the Consolidated Criteria for Reporting Qualitative Research COREQ checklist (Tong et al., 2007).

### 2.2 Participants and sampling technique

Pregnant women with normal pregnancy or with gestational complications that required hospitalization during the pandemic lockdown, and women who have given birth (birth—six weeks) during the lockdown of COVID-19 were eligible to participate. The duration of six weeks (postpartum period) was determined because it is the time when most women are still dependent on their support system, celebrate the newcomer with specific rituals, and can recall the experience very vividly. Two women who declined sharing their experience were excluded.

A total of 18 participants were selected through purposive and snowball sampling techniques from one hospital in Amman and from the community (nine pregnant or postpartum women through each technique). Snowball sampling and phone interviewing allowed the researchers to overcome geographic accessibility limitations, by reaching distant locations, which offered us a variation of experiences based on various residential areas and family structures and processes.

Six pregnant and 12 postpartum women were included in this study. At the time of the interview, the mean gestational age of the pregnant women and the mean of after birth days of the postpartum women were 31 weeks and 27.4 days, respectively. The mean age of participants was 28.7 years. Of the participants, 12 had a bachelor degree, and 11 were unemployed (66.7%, 61.1% respectively). This pregnancy was the first one for six of the participants (33.3%). Five (27.8%) of the participants had a family monthly income below the national poverty level (323 Jordanian dinars (JOD)/month per family size of 5.7 persons; International Labour Office, 2013); (1 JD = 1.41 U.S. dollars). Fourteen out of the 18 participants (77.8%) had health insurance. During pregnancy, seven of the participants (38.9%) reported health problems such as hypothyroidism, diabetes mellitus, hypertension, urinary tract infection, genital tract infections, antepartum haemorrhage, or anaemia. Four of the participants needed hospitalization due to preterm labour pain, decreased foetal movement, and gastrointestinal problems.

### 2.3 Recruitment procedures

Recruitment of participants in the hospital was facilitated by one of the authors (FT, obstetrician) who invited potential participants to participate as they were hospitalized. Recruitment from the community was facilitated by two liaison persons, a Yoga instructor and a pharmacist, who had contact with pregnant women in the community, and continued through the snowball technique.

### 2.4 Data collection procedures

Ideally, face-to-face interviews are the best method for data collection in qualitative designs (Braun & Clarke, 2013). However, and due to COVID-19 lockdown, we used phone interviews as an alternative method for data collection. According to Grbich (1999), although a researcher may lose the advantage of body language interpretations when using phone interviews, phone calls have the advantage of “being impersonal” and the possibility for the interviewee to “simply hang up” (p. 101).

After obtaining a participant’s consent for participation in a scheduled phone interview, a digital demographic survey questionnaire was completed by hospitalized participants; this form was completed after the phone interview by participants from the community. The purpose of the phone interview was to obtain a full account of the experience of being pregnant during the lockdown of COVID-19. A flexible open-ended questions guide was prepared based on our reading of the literature about COVID-19 and our familiarity with the theoretical psychological changes occurring during pregnancy and birth. Nine major interview questions and sub-questions were prepared to explore expectant mothers’ concerns, feelings, protective practices they used, and sources used to adapt with the new conditions. Example of questions in the interview guide: Please tell me about how you felt as you were pregnant and learned about COVID-19 and the lockdown? What are your major concerns during the lockdown of COVID-19? How do you compare this pregnancy with your previous one? In-depth probing questions were invited as the phone call progressed. Examples of questions raised as probing an answer: (1) can you elaborate more on your fears regarding the infection? Please walk me through your concerns about the birth journey. Can you walk me through the admission and hospitalization phases?

The principal researcher [RS], a female, skilled in qualitative research interviewing conducted all the interviews (in Arabic) to maintain consistency of tone and organization of the accounts. Being a female researcher who can understand and sense more clearly another woman’s feelings, has enabled us to capture the innate feelings and health problems of expectant mothers that are considered very sensitive, private and personal. All interviews ranging from 25 to 45 minutes were voice-recorded. Data were collected during curfew from April 30 to 21 June 2020.

### 2.5 Data analysis

Thematic analysis using Braun and Clarke (2013) eight stages of coding and analysis was used. Firstly, voice recordings were transcribed verbatim soon after each interview by one of the researchers (SE). All Arabic transcripts were translated into English by a professional translator and were checked by all the researchers (bilingual), ascertaining that the meaning was maintained precisely. Preliminary analysis of the interviews was conducted simultaneously with data collection in search for probing questions and hence further details about the experience. Reading and familiarization of data were done concurrently by all the researchers and coding was completed across the entire data set in developing themes. Our initial coding was guided by Saldana’s method for understanding social processes of human action, reaction, and interaction.In our reading, we were searching for patterns in describing the routines, rituals, rules, roles, and relationships (Saldana & Omasta, 2018). Initial assigned codes were discussed and a consensus about the codes was reached over recorded online platform meetings that lasted for hours. Codes were then condensed into provisional themes and subthemes and the relationship between them (Braun & Clarke, 2013). The researchers were aware of their personal biases as maternity health professionals, living the same crisis of the pandemic, and tried to avoid implicit biased interpretations by reaching an agreement on each code and constructed theme. Themes that were not fully saturated were probed in the next interviews until reaching a sense of saturation at around the 15th interview. Three more interviews were added to determine that no more innovative ideas were emerging.

### 2.6 Rigour and trustworthiness

Lincoln and Guba (1985) criteria were used to ensure qualitative trustworthiness. Credibility was achieved through prolonged engagement with the subject matter (Streubert & Carpenter, 2011). The researchers have engaged largely with women’s experiences during COVID-19, as we lived the same lockdown circumstances, and listened to women’s stories, and read social media platforms. Additionally, although we had phone interviews, out of the 20 pregnant women we approached for permission to conduct an interview, only two women refused participation. Participants were enthusiastic to share their experiences and fears, by talking freely to someone who is listening. Member checking was sought by contacting, by phone, four participants in soliciting their views of the credibility of the findings and our interpretations. They all confirmed that our findings and themes described the reality of their experience. In support of confirmability, nine of the participants continued to ask questions on the phone after stopping the recording. We found that participants were less self-conscious when talking on the phone, contrary to face-to-face interviews, especially that these calls were planned at a participant’s convenience. Participants were verbose by giving details of their experience and added further details that were not within the scope of the study (i.e., family issues and health questions), and many had continued asking sensitive questions about sexuality concerns even after ending the interview. Dependability was supported by triangulating our data with the content posted on social media and through our contact with HCPs who also confirmed our findings. In another aspect, we were, as physicians and nurses, directly and closely involved with the changing reactions of the public towards the lockdown and its impact on the healthcare system and its beneficiaries. Transferability was assured through offering a rich and thick description of the research context (COVID-19 lockdown) and the analysis methods we used to complete writing the themes (Creswell, 2013).

### 2.7 Ethical considerations

Ethical approval was obtained from the Academic Research Committees at the School of Nursing, The University of Jordan (#: PMs.19.13). In research work, pregnant women are considered among the vulnerable groups. Meanwhile, in critical times, pregnant women need to be heard and understood while considering their rights to voluntary participation or withdrawal from participation without reprimand. This research conforms to the ethical principles for Medical research on human beings set out in the Declaration of Helsinki (WHO, 2013)

All potential participants were informed verbally about the purpose, procedures of research, and assured of confidentiality, safety, and their rights to beneficence, non-maleficence and justice. Upon initial agreement to participate, they were contacted by phone by the principal researcher to obtain their verbal consent for participation in a phone interview. An appointment for a later prolonged phone interview was arranged based on participants’ convenience in time.

For data safety, an original copy of voice recordings, transcripts, and electronic data was saved on the principal researcher personal computer under a password. A copy with pseudonyms (serial numbers) was shared with the co-authors under strict security and a password on their computers.

## 3. Results

Three main themes underlined with five subthemes were constructed through qualitative data analysis. Examples of codes and corresponding themes are presented Table I.

**Table I.** Examples of the process of creating codes, sub themes and themes from significant statements

Significant Statements	Codes	Subtheme	Theme
“I was frightened by warning messages seen on TV, ‘no one should leave home, especially pregnant women, you have low immunity.” (Transcript 10, page 2, lines 35–36).	Media and authority control exaggerated fear feelings.		Living with fears and uncertainties amid the COVID-19 pandemic
“the world and the hospitals were closed because of lockdown; no one can go outside … I was stressed because I cannot do prenatal care to reassure myself about my baby’s health.” (Transcript 4, page 3, lines 60–62).	Lockdown and associated rules disturbing prenatal care routines.	Disruption of prenatal care and support	Lockdown disrupting the normalcy of pregnancy
“I am scared, not because of the lockdown, but because my home is far from the hospital in which I want to give birth. I want to take the epidural for pain relief, but in the nearby hospital (a public hospital), it is not administered. I am scared that I arrive late and miss the chance for an epidural.” (Transcript 2, page 1, lines 12–14).	Fear and uncertain from not fulfiling birth expectation.	Uncertainty about fulfiling birth plans	
“I have a chlorine bottle distilled with water and two hygiene bottles near the entrance. As soon as my husband comes in, he is sprayed from head to toe. He should take off his clothes by the door and everything is taken to the washing machine immediately.” (Transcript 12, page 3, lines 35–37).	Changing life style routines to be overprotective.	Taking extra precautions regarding the fear of infection.	Trying to control the chaos of life
“I used to call my physician by phone If anything happens to me. He calmed me down and told me that there was no need to leave my home and go to the hospital. He immediately prescribed medication on the phone … he also had a page on Instagram where he uploaded updates about corona transmission from mother to fetus.”(Transcript 8, page 1, lines 12–17).	Feeling reassured by switching to some alternative modalities in provision care.	Seeking reassurance from various available sources	
“ I was very afraid when the lockdown was enforced; cars’ movement was prohibited, so I immediately called my obstetrician and asked for a caesarean, not a normal vaginal delivery. I did not feel any sign of labor then … I decided to go for cesarean to feel certain, reassured, and relieved. I was afraid that the corona pandemic conditions would worsen as there were warning messages that corona would spread, and the hospitals would be closed.” (Transcript 7, page 1, lines 10–16).	Fear of the unpredictable conditions leading to Choosing elective CS.	Choosing the “unusual” for reclaiming control	

### 3.1 Living with fears and uncertainties amid the COVID-19 pandemic

Fear and uncertainty were the overwhelming feelings across all participants’ accounts. Tales were pointing to the influence of the media as increasing their fears. This sense was conveyed by one of the participants (10), who was 39 years old at 24 weeks’ gestation and a mother of four children, “I was frightened by warning messages delivered on TV, ‘no one should leave home, especially pregnant women, you have low immunity.’” Similar feelings were echoed by participant (9), who was 26 years old at 34 weeks’ gestation, as she described the media coverage about COVID-19:

We have seen what was happening in the European countries on TV and Facebook. I wondered what would happen to us when seeing Italy and America losing control in addressing this pandemic; how can we! It’s kind of the unknown. The future is vague.

The community has also contributed to increasing pregnant women’s fears by providing unsolicited advice about pregnant women’s low immunity and vulnerability. Participant (11), a pharmacist, described her customers’ reactions as “At the beginning of Corona, people’s reactions on seeing me pregnant and working was the scariest thing. They say, ‘be careful you are pregnant!’; this has frightened me tremendously.” Hospitalized participants were afraid of contact with HCPs and hypercritical of their non-adherence to COVID-19 standard precautions. A high-risk pregnant woman (10), who suffered gastrointestinal symptoms, described this situation with resentment by saying: “Some nurses were committed to masks precautions, but many were not! They had low masks, just for show-up. Nurses were in Emergency Rooms without gloves!”

Participants were afraid about contracting the infection and transmitting it to the foetus. Five participants who had pregnancy complications were reluctant to seek healthcare in fear of contracting the infection and transmitting that to their foetus. Participant (10), at 24 weeks’ gestation, was admitted to the hospital after developing severe dehydration because of delayed healthcare. She explained, “I had acute abdominal pain and diarrhea, and I was very sick … I couldn’t tolerate pain. I was dehydrated … I kept delaying seeking healthcare because I was afraid of going outside.”

Moreover, all participants were overwhelmed with messages that heralded a foetus’ vulnerability through vertical transmission. These messages provoked feelings of uncertainty, a sense of vigilance, and exaggerated precautions at home, upon hospitalization, and after discharge. Participant (13), 29 years old, a mother of two children, described her fear of transmission by saying, “I am not only worried about myself getting the infection, but also very worried that I transmit it to my baby. This is my nightmare.” In response to fear about infant’s getting infected was expressed by participant (11) who said, “If things stay like this when I give birth, I will wrap my baby, totally insulate him with covers and let nobody touch him, except me, even if that was a close relative.”

Overall, the media and the community have contributed to mothers’ confusion about their health and the health of their newborns. Messages about vulnerability have triggered feelings of uncertainty and have added to mothers’ fears.

### 3.2 Lockdown disrupting the normalcy of pregnancy

Enforced absolute lockdown has resulted in complete cessation of healthcare services in the public and private sectors. With this closure, the participants lost an essential part of their pregnancy rituals and routines and started to rethink their PNC and birth plans.

#### 3.2.1 Disruption of prenatal care and support

All perinatal care at outpatient clinics was abruptly closed in response to lockdown. Cessation of care has increased participants’ stress and fears, especially among mothers in the third trimester of pregnancy. Participant (4), 26 years old and a mother of two children, expressed her concerns as “the world and the hospitals have closed because of lockdown; no one can go outside … I was very stressed because I cannot do prenatal care to reassure myself about my baby’s health.” Moreover, high-risk participants complained about being deprived of essential healthcare services, family, and social support because of the lockdown. Participant (1), 35 years old, who gave birth at 36 weeks’ gestation to her first baby boy after five girls, described her experience as:

I have anemia and have taken the iron supplement for one month. Because of corona lockdown, I was unable to check my hemoglobin. It was difficult to do laboratory tests such as haemoglobin and urinary analysis before my cesarean section, neither was I able to do the follow-up visits in the last weeks before the operation.

Three participants delayed or did not seek PNC even after releasing curfew hours and restoring this service. Participant (14), 30 years old, with high school education and a mother of two children, postponed prenatal care for two months, she said, “I suffered a lot; I did not do a prenatal follow-up in the seventh and eighth months of pregnancy … I remained anxious, and I was never comfortable”. Similarly, participant (11), a first-time pregnant woman, said, “I did not go to my long awaited 4-D testing and I cancelled it in fear of corona”. A 24 years old participant (15) with secondary education who developed hypothyroidism during pregnancy described her experience with inadequate prenatal follow-up as she was informed that her baby had had a deformity in his feet:

During the lockdown, I could not do the prenatal follow-up. All clinics were closed. I missed the seventh, eighth, and ninth months’ visits. I was anxious; I wanted to be reassured about my baby’s health … I was shocked when I learned about his feet problem … I was furious when the nurse told me that.

Even the doctors had to modify the usual pattern of providing PNC by asking their patients to call them by phone rather than visiting in clinic or by advising them against doing the visit. Participant (16), a 22-year old, said,

My doctor told me to stop my antenatal visits … the doctor said, ‘you may tell me all about your health on the phone, do not come for your safety because Corona affects pregnant women and young children more than others.

Participants in this study showed concern about missing PNC due to lockdown and closures, however their worries about contracting the infection have stopped them from resuming visits even after lockdown measures were released. For some participants, a phone contact with their doctor was a good alternative to alleviate their worries about missing PNC.

#### 3.2.2 Uncertainty about fulfiling birth plans

The participants expressed their concerns about the uncertainties of their birth plans during the lockdown. They were worried about not making it to the hospital on time. Participant (5), 30 years old and a mother of one child, said

I am stressed because we were not sure about going by our own car to the hospital during the lockdown. I don’t like to call the Civil Defense Service because they may come late. I don’t know what to expect!

A more difficult feeling was conveyed by ten participants who were afraid of giving birth without a companion, especially without their mothers, and all alone. Participant (14), who had a previous experience in induced labour, said

During birth, no companion was allowed, not even my husband. It was a very annoying situation … my last birth was induced, painful and tiresome … I was unable to change my position or to move without my mother’s and sister’s help.

Participant 3 said “I was worried because I wanted somebody to be with me in labor. Visiting was a problem”. Seven participants were concerned about losing control over their birth plans because of mobility restrictions. These concerns included not receiving the selected pain relief medication, the hospital of birth, or her obstetrician. Participant (2), who lived in a different governorate from the hospital of her choice, described her worries as

I am scared, not because of the lockdown, but because my home is far from the hospital where I want to give birth. I want to take the epidural for pain relief, but it is not administered in the nearby hospital (a public hospital). I am scared that I arrive late and miss the chance for an epidural.

A 38 weeks’ pregnant participant (6), a mother of one child, described her uncertainties as she started to feel the first signs of labour during the complete lockdown:

I was afraid that the anesthesiologist would not be on time. I wanted a particular person; I knew nothing. I was afraid that he would not be available. The second thing was the stem cells; they are my baby’s future.

Ten of the participants were sad and disappointed because they did not finish preparations, such as clothing and furniture for the newborn’s arrival. Participant (12), with a history of perinatal loss, clarified this by saying:

Because of the lockdown, I had nothing prepared for my baby … I have some clothes and baby supplies from the first pregnancy … before lockdown, I planned to get this stuff prepared during the last trimester. But now, I do not have a crib nor clothes for my baby. My sister had small kids, so I had to borrow clothes from her, which disappointed me.

In brief, the participants in this study had many challenges to think about as they were waiting for birth. Most importantly, they were concerned about reaching the hospital, not having a companion, not meeting their expected birth plans, and not completing preparations for the newcomer.

### 3.3 Trying to control the chaos of life

As participants lived the COVID-19 pandemic chaos with fear and uncertainty, they tried to mitigate that by taking actions on their own. Taking extra transmission prevention precautions, seeking reassurance from various available sources, and choosing the unusual were the three major strategies that increased their sense of control over their fears, uncertainties, and confusion.

#### 3.3.1 Taking extra precautions in fear of infection

Sixteen participants in this study regulated strict safety precautions in response to the threat of being infected with COVID-19. Precautionary measures included exaggerated disinfection in their homes and work, setting new rules for the family, and limiting socialization. Participant (4) eloquently described her priority in using disinfection measures at home: “Disinfectants have become more important than bread.” Rules of disinfection were also applied to husbands and children. Participant (12), said

I have a chlorine bottle distilled with water and two hygiene bottles near the entrance. As soon as my husband comes in, he is sprayed from head to toe. He should take off his clothes by the door, and everything is taken to the washing machine immediately.

Disinfection measures were irritating to the participant’s (11) husband creating tension in family relationships. She narrated her husband’s complaints about her exaggerated and obsessive cleanliness, which led to marital tension. She said

As he came into the house, I asked him to put all his clothes aside. I noticed later that this had irritated him tremendously. One day, he grumbled and said that this subject (infection) had overtaken my whole day’s talk.

Five participants also restricted their children’s whereabouts. They have forbidden them from outdoor playing with other children. Participant (10) said, “I was afraid to let my children go out. I imprisoned them at home, not even at the front yard, I was afraid of the air breeze.” This kind of mother’s control has resulted in children’s frustration and complaint about interfering with their usual play activities. Participant (10), a mother of four children, said: “my children complained about restricting their play with their friends in the front yard.”

Furthermore, fear of the COVID-19 transmission has implied setting new rules and practices for avoiding socialization. Participant (9) described her experience of limited personal and social activities in the following words: “I did not leave home, nor let anyone go out or visit me during the lockdown. I enforced lockdown strictly. My parents are across the street, but none could visit.”

Strict disinfection precautions have also affected working participants’ relationships with customers at work. They felt worried and uncomfortable when providing service to customers as described by the participant (11, a pharmacist):

I became fearful in dealing with any human being, even my colleagues at work. I was afraid to use the restroom in the pharmacy (drugstore), afraid of going home, nervous all the time. I was nervous when customers came in and asked them to stay away, take their purchases quickly, and leave.

One participant (17) who was a nurse on night or day shifts described how the supervisor and her colleagues limited her exposure to COVID-19..

They cared for me by giving me fewer hours at work. For example, they helped me to cover the beginning of the shift and to leave early. They helped me to take days off when possible. They would call me at night telling me not to come to work the next day and that someone else was covering for me. They were very cooperative.

Lockdown restrictions and fear of infection have certainly implied modifications of the participants’ lifestyle and behaviours which probably have imposed its negative consequences on family relationships and work environment.

#### 3.3.2 Seeking reassurance from various available sources

Along with taking serious disinfection measures, participants attempted to equip themselves with the latest governmental reports regarding lockdown of COVID-19 and relied on one’s knowledge and others’ experiences. Because of the cessation of PNC, mothers had limited trusted sources of health information. Electronic and online sources replaced formal PNC. For six participants, phone applications and social media platforms were the only references to answer their questions and help them manage their health concerns. Participant (9) said,

Now, I use an application on my phone. I enter my inquiry about what a pregnant woman should have or feel at this stage of pregnancy and compare that with my symptoms; this is how I get reassurance about my pregnancy.

Participant (10) referred to social media platforms as a reliable source about pregnancy and COVID-19; she said, “My information source is Facebook. From there, I learned from a woman with COVID-19 who shared her experience of giving birth during COVID-19.” Participant (8), who attended a private clinic, had a more formal and trusted online source of information. She described a continued contact with her physician over the phone and social media sources:

I used to call my physician when I needed. He reassured me and said that there was no need to go to the hospital to get the drug. He immediately prescribed medication on the phone … he also had a page on Instagram where he uploaded updates about corona transmission from mother to fetus.

Another participant (17), who gave birth for the third time and worked as a nurse in a hospital, suggested doing check-ups over the phone or via video calling to reassure pregnant women about their foetuses’ health when they cannot reach the hospital:

Doctors can answer questions via phone; they can assess a pregnant woman for problems using video calls. It will comfort the patient psychologically and help them decide if that was an emergency requiring a hospital visit. Doctors should allow patients to reach them by phone or video meetings, no excuses with the available technology.

Six participants depended on their previous pregnancy experience and foetal movements sensing to assure themselves about the foetus condition as they were deprived of formal PNC. Participant (9) stated:

During the lockdown, I had no prenatal visits as I stayed at home. I felt nothing wrong during this period, and the fetal movements were good. I had little fear; if I feel the baby moves, I know my condition is okay.

On a further note, two participants who were nurses themselves or had a family member who was a healthcare provider were less worried about their health condition during the lockdown. Participant (18), 27 years old, gave birth for the third time, said, “my brother is a doctor. He gave us information about Corona and how to deal with it.” Participant (2) and her husband were practicing nurses; she described her experience with pregnancy complications as:

Regarding prenatal visits, I am a nurse; I know everything. I know when the fetal movement and other signs are good and feel reassured. I know when things go wrong and need to see a physician …. My husband helped me a lot during this period. He withdrew a blood sample for me at home and took it to the hospital for hemoglobin testing because I was anemic in the ninth month …. I had frequent episodes of hypotension and chest pain …. We know these signs because I experienced these in my previous pregnancy.

In the uncertainties of the lockdown restrictions, the participants had to use several alternative sources in search for help and reassurance. Results have emphasized the importance of the new technology (i.e., social media formal and informal), being a health practitioner, and previous personal experiences of birth as alternatives to institutional healthcare services.

#### 3.3.3 Choosing the “unusual” for reclaiming control

Once the pandemic had spread, and lockdown restrictions were enforced, expectant mothers made profound decisions anticipating more critical conditions. Gradually, and as their fears escalated, they felt the immediate need to protect themselves and their foetus from contracting the infection and to restore some control over their pregnancy and birth. Participant (7), a first-time pregnant woman, elected to go for caesarean birth although this was not medically indicated. She chose to go for caesarean to make her birth more predictable and under her control. She explained:

I was very afraid when the lockdown was enforced; cars’ movement was prohibited, so I immediately called my obstetrician and asked for a cesarean, not a normal vaginal delivery. I did not feel any sign of labor then … I decided to go for a cesarean to feel certain, reassured, and relieved. I was afraid that the pandemic conditions would worsen as there were warning messages that Corona would spread largely, and the hospitals would be closed.

Participant (1), who gave birth at 36 weeks’ gestation contacted her doctor asking for a caesarean birth, said, “I was so scared which made me decide to go for a cesarean and get over with pregnancy. I made this decision as they announced complete lockdown and no car movements.”

Because of these unpredictable conditions, participant (9) had even made longer-term decisions to protect her newborn after birth by saying: “The first thing I will do is to cancel people’s visits to see my baby, no baby gifts, nothing like this should happen.”

In brief, this theme reflects the fear of the unpredictability of COVID-19 conditions. Pregnant mothers had to integrate exaggerated changes of disinfection and social distancing into their lifestyle to protect themselves and their infants. They also looked for possible means to obtain information that reassures them of the progress of their pregnancy, as well as to choose the unplanned alternatives, such as caesarean birth or separation from their support system for the sake of instilling certainty and safety of their pregnancy, birth, and the newborn’s well-being.

## 4. Discussion

**Figure 1.**
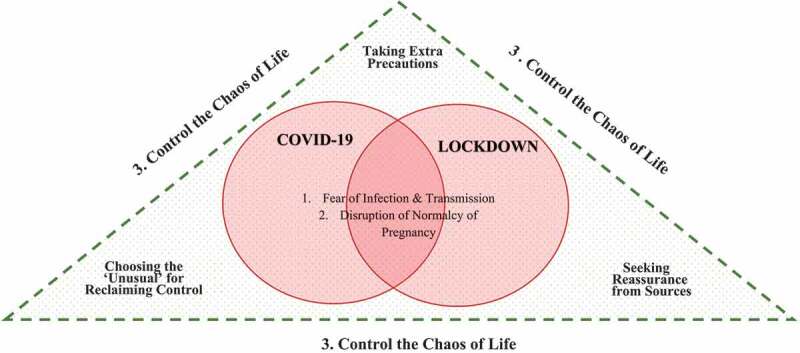
Model of emerging interactive themes.

In this study, we sought to explore the interface of being pregnant and living the unusual circumstances of the lockdown of COVID-19. The findings show the development of three main themes: (1) Living with fears and worries amid the COVID-19 pandemic, (2) Lockdown disrupting the normalcy of pregnancy, and (3) Trying to control the chaos of life. Five subthemes underlie the latter two main themes. Themes and subthemes are shown in Figure 1.

The findings of this study showed that during the pandemic the participants had to handle the uncertainties of pregnancy complicated by fears of contracting the infection and transmitting it to their foetuses. The lockdown—closures and movement restrictions—have considerably added to expectant mothers’ fears and uncertainties.

In line with the pervasive literature that explored the emotional status of pregnant women during COVID-19, our study offered further evidence about the fears and anxieties that expectant mothers experienced (Mizrak Sahin & Kabakci, 2021; Mortazavi & Ghardashi, 2021). Congruent with previous reports (Karavadra et al., 2020; Mizrak Sahin & Kabakci, 2021; Mortazavi & Ghardashi, 2021; Tadesse, 2020), this study conveyed participants’ fears of the risk of viral transmission affecting themselves or their foetuses when attending health institutions. Expectant mothers’ perception of the hospital environment as risky was also found as preventing mothers from receiving care (Karavadra et al., 2020). In a study that examined predictors of fear of COVID-19, the perceived risk of the virus for oneself and loved ones in particular, was the best predictor of more fear of COVID-19 (Mertens et al., 2020).

In Jordan, official news coverage and social media have played a major part in amplifying expectant mothers’ fears. A relevant explanation is borrowed from the WHO Director-General Ghebreyesus who declared that “a global epidemic of misinformation—spreading rapidly through social media platforms and other outlets—poses a serious problem for public health. We’re not just fighting an epidemic; we’re fighting an infodemic” (Zarocostas, 2020, p. 676). In this sense, we saw mothers’ increasing fear as they heard the warnings and the news of more intensive precautions, restrictions, and an increasing number of cases around the world. Considering how the pandemic was framed in the media, and the words chosen in the coverage can either alleviate or exaggerate people’s perceptions and responses (Ogbodo et al., 2020). Through media channels, the government authorities of Jordan provided daily updates on the situation in the country. Although these messages were in the best interest of the public to reduce viral transmission, they were scary and resulted in an exaggerated response by our participants. Although fear of the pandemic can activate protective response, the exaggerated fear of the pandemic may be more harmful than the disease itself (Ren et al., 2020).

In the second theme, strict lockdown contributed to participants’ worries because of the cessation of perinatal care and reconsideration of birth plans. Prenatal care that is adopted by pregnant women as a vital pregnancy ritual, was cancelled during the lockdown in Jordan. For safeguarding purposes, mothers were advised to stay at home and were asked to call the Civil Defence in case of an emergency. During the pandemic, there were fewer PNC visits, a strained healthcare infrastructure, and harmful policies implemented without real evidence (Kotlar et al., 2021). Prenatal care services were immensely affected in many countries and had negatively influenced women’s health. In the United States, cancelling or rescheduling PNC were the main predictors for high preparedness stress and perinatal infection stress among nine other predictors (Preis et al., 2020). Disruption of PNC routines and social lives was negatively affecting pregnant women’s emotional health in Turkey (Mizrak Sahin & Kabakci, 2021).

In response to the interruption of PNC, literature shows suggestions of alternative methods to replace the lost care and meet mothers’ needs while keeping them safe (Holcomb et al., 2020; Kotlar et al., 2021; Pasadino et al., 2020). Telemedicine, virtual counselling and education, and remote support networks were started in many countries, such as in the United Kingdom (Karavadra et al., 2020), United States (Pasadino et al., 2020), and Poland (Jakubowski et al., 2021). In Jordan, the healthcare system’s efforts were directed towards controlling the spread of infection generally, and no specific alternative plans were devised to replace the cancelled regular PNC.

The study showed that lockdown was a major worrying factor for expectant mothers. Curfew hours were highly emphasized with warning sirens announcing the time beginning and enforced by the police when ignored. Our participants perceived this strict lockdown as emotionally straining and were uncertain about whether upon pending labour, they will be able to reach the hospital they wanted, by their car, on time, managed by their obstetrician, received the pain medication of choice, and surrounded by their family. Due to severe lockdown restrictions, expectant mothers were deprived of family support and the joy of preparations for their newborns. This result is novel as we found no literature that described such a stressful experience during the pandemic as lockdown restrictions were one of the strictest in the world.

In response to the fear of the pandemic, the lockdown, and the media influence, expectant mothers had to act, which forms the third focus/theme of this discussion. With the prevailing sense of uncertainty and chaos at the time of lockdown, our participants tried to readjust to the new situation of increased vulnerability, loss of PNC and interrupted birth plans, and lack of information about the impending situation. Expectant mothers followed all rules of disinfection and social distancing with exaggeration. These protective measures were recommended; however, the exaggerated use was a sign of the stress they were living. Disinfection and distancing became major parts of their life that had a damaging effect on their relationship with family and others in the workplace. Emotional support is most needed during pregnancy, and lack of that may result in depression and mental health issues that may extend to the postpartum period (Chivers et al., 2020; Ollivier et al., 2021).

Additionally, and due to the loss of PNC and as a sign of worry about the looming conditions they were living, expectant mothers sought information from all available sources to reassure themselves about their pregnancy progress and foetal well-being. They sought information from non-official electronic sources, used their previous experience of pregnancy, and their knowledge if they were nurses themselves. According to Lazarus and Folkman’s transactional theory of stress and coping, women in this study were adaptive; they used some problem-focused and healthy coping strategies, such as taking control of the stress, seeking information or assistance, and removing oneself from the stressful situation (Carroll, 2013).

Regarding the third theme in attempts of maintaining some certainty and control, findings showed that mothers have used three strategies to be in control of the uncertainties of COVID-19. One of the significant strategies was requesting a caesarean birth while it was not medically indicated. The recent worldwide literature reports increasing rates of caesarean births that are performed according to women’s requests. The most commonly reported reasons behind a woman’s choice of a caesarean birth are mainly related to fear of birth and labour pain, negative previous experience, avoidance of pelvic prolapse/urinary incontinence, and influence of others (Jenabi et al., 2020; O’donovan & O’donovan, 2017). However, in our study, participants chose to undergo a caesarean birth for a different reason without a clear medical or obstetrical indication. This case of *asking for a caesarean* appeared unusual or different because it was provoked by fear of giving birth under the uncontrollable conditions of the pandemic instead of a normal vaginal birth according to their birth plan.

In Jordan, according to JPFHS, the overall rate of caesarean births was 26% (Department of Statistics (DOS) and ICF, 2019). A study by Salem (2021) revealed an increasing trend of caesarean births as it reached 31.8% in 2017 compared 6.0% in 1983. According to this study, the most common indications for caesarean births were a previous caesarean (33.6%), abnormal presentation (20.3%), and patient request (16%; Salem, 2021). The latter finding (16%) is limited to one private hospital in Amman, and thus does not represent mothers request for caesarean in the entire population. In another study (Batieha et al.’s (2017), the rate of emergency and elective caesarean sections was 13.2% and 15.9% respectively; elective caesareans were mostly performed for medical indications such as scarred uterus (59.4%) and abnormal presentation (7.9%). The elective caesareans performed because of mothers’ request accounted for 5.6% only. In our study, and beyond the medically related factors, some participants have elected to bypass unpredictable labour by choosing a caesarean birth, a result that is consistent with the existing evidence. Limited access to support, lack of a companion at the time of birth have increased expectant mothers’ stress (Jago et al., 2020; Kotlar et al., 2021), and those who are afraid and anxious about their foetus during the pandemic opted to terminate their pregnancies prematurely or by a caesarean birth (Rashidi Fakari & Simbar, 2020). The rate of preterm birth and caesarean birth during the pandemic were found to be considerably higher than the international averages (Matar et al., 2021).

In another unusual action taken by mothers in response to COVID-19, and against the familiar social norms of after birth traditions and expected family support, mothers have planned to restrict their social interactions. In Jordan and most Muslim cultures, birth of a baby is usually celebrated with a specific ritual known as Aqiqa, a celebration that is comparable to baby shower in the West. Aqiqa is a recommended Islamic tradition to sacrifice an animal (usually a sheep) within the first week of birth in an expression of gratitude to Allah for the blessings for having a baby. On this occasion, all family members and close friends are invited to a special dinner to celebrate the arrival of the baby. Celebrating this significant ritual was cancelled during the COVID-19 pandemic for the sake of their safety and the safety of their newborns.

### 4.1 Strengths and limitations

To the best of our knowledge, this is the first descriptive qualitative study that explored expectant mothers’ experience of the lockdown of COVID-19 pandemic in Jordan. Data were obtained from expectant mothers who genuinely wanted to share their experience through phone interviews and completed before lockdown restrictions were released. The study was limited to phone interviews and thus we may have lost the added richness of non-verbal cues obtained through face-to-face interviews. Additionally, the researchers were limited in data collection time as the aim was to capture the experience while women were still living the lockdown constraints. We were also limited by our own immobility to access more hospitals and healthcare centres or to have access to a larger sample and variation of expectant mothers which might have resulted in some selection bias of the sample. Nevertheless, we believe that within these constraints, we were able capture the essence of expectant mothers’ experience during this pandemic.

## 5. Conclusions

The findings of this study gave voice to pregnant women in disclosing their feelings of fear and uncertainty in the time of their greatest need for support. Pregnant women lived the uncertainties of COVID-19 transmission with its risks on their health and their infants, and the lockdown restrictions depriving them of their routine PNC and the predictability of birth plans. To maintain some balance in their lives, responses such as seeking knowledge when the formal source was lacking, making decisions regarding their birth, and the use of exaggerated infection precautions to protect themselves and their unborn were captured. The investment in listening to pregnant women’s needs and concerns is worthy and critical as we contribute to the well-being of mothers and their newborns.

The study findings portrayed the fragility of the healthcare systems and lack of preparedness in times of emergency where pregnant women could be the first victims. The governmental authorities have handled the emergent COVID-19 and lockdown considering the bio-medical urgency for controlling the spread of infection irrespective of women’s reproductive and gestational health needs that are routinely regulated in the healthcare system. The findings revealed the disconnection between the beneficiaries of pre-and postpartum services from the policy makers’ and HCPs’ agendas for strategic planning. Government authorities have made their best efforts to control the spread of the pandemic, however, a comprehensive strategy that considers all components of health: physical, mental, social, and spiritual needs of pregnant women were lacking.

## 6. Implications

The sudden pandemic and lockdown were eye-openers to assess our healthcare system readiness and response in a time of crisis. Governmental, institutional, and individual strategies should be established to ensure that everybody including expectant mothers can receive required services during a crisis. In case of a pandemic, governments need to consider the specific needs of women and children when imposing lockdown restrictions. The main principles for policy actions must include a comprehensive well-coordinated, balanced, responsive management of care and governance by developing alternative plans to maintain the continuity of essential emergency services. To maintain continuity of PNC during closures, it is suggested that the government have a plan for designating specific locations/centres/ mobile clinics as referral sites for mothers in need for routine PNC.

Healthcare institutions must be ready for appropriate alternative actions. HCPs must emphasize the continuity of PNC and devise alternative innovative plans in emergencies. Educational platforms, virtual learning and consultations, mobile applications, and /or hotline for emergencies are crucial to avoid emotional stress, depression, and physical consequences in a time of crisis. Health team workgroups that use a family-centred approach in providing care during crises is recommended. At the individual level, although expectant mothers had limited mobility during COVID-19 this should not be a factor in limiting the support system she should receive from her spouse and significant others (i.e., extended family). Additionally, and due to the advancement of technology, pregnant women must have immediate access to the updates of medical science besides maintaining medical and social support (i.e., friends, doctors, family) through possible alternative means.
